# Modern Sociology

**Published:** 1901-09-07

**Authors:** 


### Modern Sociology.

### THE CASE OF THE SHOP ASSISTANT.

By a Correspondent.

### I.—Hours.

A certain famous linendraper, taking a day's holiday after "twice ten tedious years," was delayed at the last moment by three customers whose minds were ill to suit. More than a hundred years have passed, but the three customers continue to come in at the last moment, and the quarter of a million of shop assistants, the descendants of the apprentices of Gilpin's day, are the sufferers by reason of the long hours they have to work. For, difficult as it is to generalise on a subject so vast and complex as that of the modern conditions of shop life, it is safe to say that, as a rule, the hours are far too long.

What are the hours worked? There are some few good houses of business where six o'clock is the closing time, but for every one of these there are hundreds that keep open long after lighted gas, heat, dust, and combined human breaths have created an atmosphere highly detrimental to health. It is the cutting shops, aiming at large profits, that are the chief sinners; and these, broadly speaking, keep their doors open as long as there are customers to buy. These are the hours recently given for the rank and file of shops in working-class neighbourhoods:—Close at 9.30 on five nights, and 11.30 on Saturday, no half holiday; 10 on five nights, and 11.30 on Saturday; 10 or 10.30 on five nights, and 12.30 on Saturday.[1]

"Elders of churches," said an Edinburgh witness before the Lords' Select Committee the other day, "close at 12.25 on Sunday morning; and the tendency is for other shops to drift after those that are open late."

But even supposing the generality of shop assistants only worked the maximum number of hours allowed to young persons under the Shop Hours Act, *i.e.* 74, including meal times, in any one week, how does this compare—to consider for the moment women assistants only—with the hours worked by dressmakers and milliners in the workrooms belonging to the shop where the dresses and hats are sold? It will readily be seen that the work-girl is far better off than her sister serving behind the counter. The total hours of work permitted in non-textile factories are 60 per week, exclusive of meal-times. Overtime is only allowed on 48 days in a year in factories and workshops where "the making-up of any article of wearing apparel" is carried on. But long after the work-girls have gone home, the unfortunate shop assistant may be kept behind the counter, the hands of the clock may drag, and her head and back may ache, but as long as the employer—or the company—chooses to keep her there he may do so, as soon as she has turned eighteen, and has ceased to be a young person in the eyes of the law. "One of the masters under whom I was apprenticed," says "A Manager," writing in the *Economic Review*, "made a habit of walking round to see if the other drapers were open, or if any people were still about, before giving the order to close." On both sexes the long hours press heavily.

Are these long hours necessary? Would any class of the community suffer by their being shortened? If any, it would be the wage-earning classes, who, we are often told, do not receive their money early enough to shop in the fore part of the day. But is this true? Fortunately, we have among us a body of working people, numbering hundreds of thousands, who have settled the question for themselves; they were referred to by the representative of the Edinburgh and Leith Shop Assistants' Union before the Lords' Committee. "The Co-operative Stores," he said, "close early; and we are of opinion that if they can do it, private traders can." Here are the hours of the Oldham Industrial Co-operative Society, as given by Mr. Frank Hardern in his evidence before the Shop Hours Committee of 1892:—Monday, Wednesday and Thursday, 8 a.m. to 12 noon, 1 p.m. to 8 p.m.; Tuesday, 8 a.m. to 12 noon, half holiday; Friday, 8 a.m. to 12 noon, 1 p.m. to 9 p.m.; Saturday, 8 a.m. to 12 noon, 1 p.m. to 6 p.m. It will be observed that the store is closed from 12 to 1 in the middle of the day, when the assistants go away and have their midday meal and return like other workmen, fresh for the afternoon's work. Mr. Hardern also gave particulars of twelve other great societies in the neighbourhood, six of them doing a trade of a quarter of a million each in the year, and showed that the average week was about 56 hours, 58 hours being the highest. Glasgow and the suburbs average 57 hours, Stratford 60, and Woolwich 60½ hours per week. It was stated at the Co-operative Congress, 1901, that the average hours were 55½ for England and Wales, and 54½ for Scotland.

The Women's Co-operative Guild[2] recently made inquiries into the hours worked by women in the stores; the returns refer to 530 assistants in 104 stores in all parts of England. Very late hours are almost unknown; 9 being the latest hour in many, even on the late night, and a good many close at 8.30 even then. Of the north country stores some have a 48 hours week, and not one exceeds 54 hours excluding meal times. Another inquiry, just made, elicited the statement from 72 branches of the Guild (comprising a membership of over 5,300 working women) that their members found no inconvenience in shopping between the hours at which their stores were open, while 62 were of opinion that late shopping was more of a habit than a necessity. A London representative of the Guild, well acquainted with working-class life, gave evidence to this effect before the Lords' Committee the other day. She said that the motives which led the Guild to desire earlier shopping were consideration for the health of the assistants and their need for leisure and education. The Rev. J. W. Horsley, of St. Peter's, Walworth, after careful inquiries and observation in South London, said that late shopping was not necessary. Wages were paid on Friday, and where they were not, the men could draw what they wanted beforehand, and the shopping could be done early on Saturday.

Most valuable is the testimony of the three hundred medical men who in 1888 petitioned Parliament to enact the Early Closing Bill. "Such prolonged hours of labour," they said, "are grievously injurious to health." Sir William MacCormac recently told the Lords' Committee that he considered standing for 12 hours exceedingly hard labour; and Sir William Church said there were certain forms of weakness and disease which were extremely aggravated by long hours.

There are three forces which might be brought to bear on the evil: (1) The public might combine, as in the American Consumers' League, to shop early; (2) The assistants (though one can hardly picture such a consummation) might strike; and (3) Parliament might intervene. We purposely omit voluntary early closing; for as a force it has been found absolutely ineffective; though it has done something towards educating public opinion.

[1] *Life in the Shop*, reprinted from the "Daily Chronicle."

[2] The Guild has a membership of 13,000 women, in 285 branches.

